# Indirect genetic effects of siblings

**DOI:** 10.1111/jcpp.70130

**Published:** 2026-02-12

**Authors:** Mathias Valstad, Espen Moen Eilertsen, Ziada Ayorech, Rosa Cheesman, Perline Demange, Nikolai Haahjem Eftedal, Alexandra Havdahl, Eivind Ystrom

**Affiliations:** ^1^ Department of Psychology, PROMENTA Research Center University of Oslo Oslo Norway; ^2^ PsychGen Centre for Genetic Epidemiology and Mental Health Norwegian Institute of Public Health Oslo Norway; ^3^ Research Department Lovisenberg Diakonale Hospital Oslo Norway; ^4^ Centre for Research on Equality in Education, Faculty of Educational Sciences University of Oslo Oslo Norway

**Keywords:** ADHD, educational performance, indirect genetic effects, siblings, genome‐based maximum likelihood, polygenic indices, MoBa

## Abstract

**Background:**

Within‐family designs are increasingly used to decompose genotype–trait associations into direct and indirect genetic effects. Many such designs, including trio designs or within‐sibship designs, assume an absence of sibling indirect genetic effects.

**Methods:**

We expand two well‐known molecular genetic within‐family designs, one variance component (genome‐based restricted maximum likelihood) and one trait‐based (structural equation modeling with polygenic indices), to estimate sibling indirect genetic effects, along with direct genetic effects. We link the Norwegian Mother, Father, and Child Cohort Study (MoBa) to Norway's national education database to model genetic effects on national standardized testing results at ages 10, 13, and 14, and on parent‐rated attention‐deficit hyperactivity disorder (ADHD) symptoms at ages 3 and 8 in up to 15,971 genotyped and phenotyped siblings.

**Results:**

Estimates of direct and indirect genetic effects from the genome‐based restricted maximum likelihood and the structural equation modeling with polygenic indices approaches converge, albeit with the variance component estimates typically an order of magnitude greater than the trait‐based estimates. We observe no indirect genetic effects of siblings on educational performance at any age, and only slightly negative indirect genetic effects of siblings on ADHD symptoms at age 3. We argue that the latter effect might reflect parental contrasting ratings.

**Conclusions:**

The results suggest that within‐family models of educational performance are unlikely to be drastically biased by an assumption of absent sibling indirect genetic effects. Combining trait‐based analyses with variance component analyses can benefit understanding of indirect genetic effects, especially when the effects are not specific to a particular mechanism.

## Introduction

Genotypes and traits covary because of direct genetic effects, indirect genetic effects, and population structure (Young, Benonisdottir, Przeworski, & Kong, 2019). Genetic studies have shown that indirect genetic effects, that is, the component of genotype–trait associations due to other genotypes than the proband's own, like those of biological parents, might be substantial (Bijma, 2014; Kong et al., 2018; McAdam, Garant, & Wilson, 2014). This has raised questions about the interpretability of results from standard genome‐wide association studies (GWAS) beyond prediction (Young et al., 2019) and has led to prolific methodological development combining molecular genetics with family structure to separate direct and indirect genetic effects on human traits (Cheesman, Ayorech, Eilertsen, & Ystrom, 2023). Such methods, including within‐sibship GWAS (Howe et al., 2022), structural equation models with polygenic indices (SEM‐PGIs; Balbona, Kim, & Keller, 2021; Kong et al., 2018), within‐ versus between‐family polygenic prediction models (Selzam et al., 2019), or genetic relatedness‐based models (Eaves, Pourcain, Smith, York, & Evans, 2014; Eilertsen et al., 2021, 2022), aim to isolate direct genetic effects or investigate indirect genetic effects of parents in their own right. Regardless, correct estimates of both direct genetic effects and parental indirect genetic effects typically depend on the absence of sibling indirect genetic effects (Demange et al., 2022; Howe et al., 2022; Wang et al., 2021; Young et al., 2019). This is because genetic variants not transmitted from a parent to a child could still be transmitted to the child's sibling, who in turn might affect the child's trait. The consequence of this caveat is a need to (a) further develop within‐family methods to account for sibling indirect genetic effects, and (b) establish the magnitude of sibling indirect genetic effects for traits of interest to researchers studying gene–trait associations.

Educational performance (Demange et al., 2022; Wang et al., 2021) and attention‐deficit hyperactivity disorder (ADHD; Eilertsen et al., 2022; Hegemann et al., 2024; Jami et al., 2023; Martin et al., 2023; Pingault et al., 2023) are two behavioral traits of widespread interest to researchers studying direct and indirect genetic effects. This interest is due in part to the psychological salience of these traits and their impact on important outcomes such as income and mortality (Breen & Karlson, 2014; Kuja‐Halkola, Lichtenstein, D'Onofrio, & Larsson, 2015; Liu, 2004). They are, however, also heritable traits where siblings can potentially exert relevant influence on each other. Transmission of traits between siblings has been operationalized as ‘sibling spillover effects’ in economics/epidemiology (Black et al., 2021; Breining, 2014; Fletcher, Hair, & Wolfe, 2012; Mallinson & Elwert, 2022; Nicoletti & Rabe, 2019) and ‘sibling interactions’ in behavior genetics (Boomsma, Everitt, & Howell, 2005; Rietveld, Posthuma, Dolan, & Boomsma, 2003; Simonoff et al., 1998). Broadly, economists have identified sibling spillovers influencing educational performance and related traits (Black et al., 2021; Breining, 2014; Fletcher et al., 2012; Mallinson & Elwert, 2022; Nicoletti & Rabe, 2019), while results from behavior genetics have suggested that the low dizygotic twin correlations in reported ADHD symptoms (Nadder, Silberg, Eaves, Maes, & Meyer, 1998) might not be due to genuine negative sibling interactions, but rather to parental rating bias (Simonoff et al., 1998). Nevertheless, molecular genetic studies of sibling indirect genetic effects on educational performance or ADHD (or any other trait for that matter) are scarce. Therefore, the magnitude of sibling indirect genetic effects for these traits is not known. By extension, it is also unclear to what extent recently developed methods combining molecular genetics with family structure (Balbona et al., 2021; Eaves et al., 2014; Eilertsen et al., 2021, 2022; Howe et al., 2022; Kong et al., 2018) could still be biased when indirect genetic effects of siblings are not explicitly modeled.

Generally, indirect genetic effects can be analyzed within either a trait‐based or a variance component framework (Bijma, 2014). Trait‐based models operationalize indirect genetic effects as effects of trait‐related values for parents or siblings of the target person, that is, a PGI. They can detect indirect genetic effects that work through phenotypic processes related to the PGI, but not through other potential phenotypic processes (Bijma, 2014), and are therefore ‘mechanism‐dependent’. In contrast, variance component models partition the index person's trait variance into direct and indirect genetic effects according to a structuring mechanism such as the number of siblings or relatedness of parents from different families. The result is that variance component frameworks can detect indirect genetic effects through any phenotypic process in the person, or, alternatively, rule out the existence of an indirect genetic effect altogether; they are therefore ‘mechanism‐independent’. That is, if a sibling indirect genetic effect is mechanism‐independent, it operates through the phenotype of the sibling and the set of potential phenotypes in the sibling through which it operates is not restricted by a PGI. In the following, we describe extensions, with sibling indirect genetic effects added, of two well‐known methods for decomposing gene–trait covariances into direct and indirect genetic effects, one variance component (Eilertsen et al., 2021) and the other trait‐based (Kong et al., 2018). Both methods are used to decompose variance in educational performance and ADHD symptomatology.

**Figure 1:**
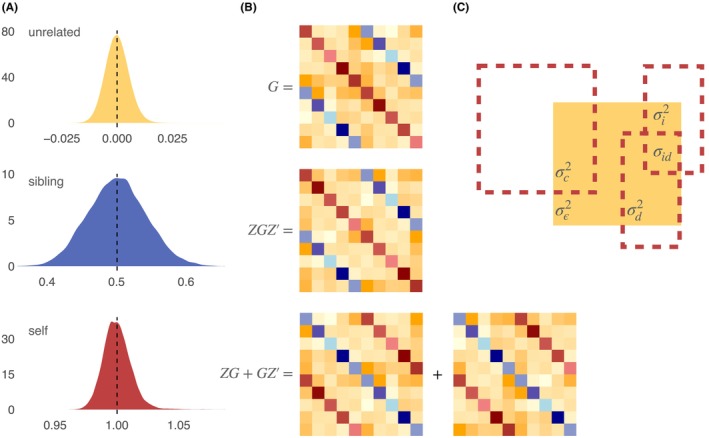
(A) Densities of genetic covariance between unrelated individuals (yellow), siblings (blue), and selves (i.e. genetic variance, red). The star of the GREML show is the random genetic covariance between unrelated individuals (yellow). Since this genetic covariance is random, an increased phenotypic similarity among individuals with greater genetic similarity must be due to the effect of genes. But whose genes? (B) The genetic relatedness matrix, G, of five simulated sibling pairs demonstrating the genetic covariance between unrelated individuals (yellow), siblings (blue), and selves (i.e. genetic variance, red). The sibling matrix, Z, which indicates with a one or a zero whether a pair of individuals are siblings, would have ones where G is blue, and zeros otherwise. ${\mathrm{ZGZ}}^{\prime }$ gives us a matrix where the *i*, *j*th element is the genetic covariance between the sibling of *i* and the sibling of *j*. When *i* and *j* are siblings, this is the same as the genetic covariance between *i* and *j*. When *i* = *j*, this is the same as the genetic variance of *i*'s sibling. Hence, in the specification $\mathrm{Cov}\left(y\right)=\sigma_{i}^{2}{\mathrm{ZGZ}}^{\prime }$, the parameter $\sigma_{i}^{2}$ reflects the association between *i*, *j*'s phenotypic similarity and their siblings' genetic similarity. Moreover, in the specification $\mathrm{Cov}\left(y\right)=\sigma_{d}^{2}G+\sigma_{i}^{2}{\mathrm{ZGZ}}^{\prime }$, the parameter $\sigma_{i}^{2}$ reflects the extent to which this association goes beyond the association between *i*,*j*'s phenotypic similarity and their own genetic similarity. (C) Phenotypic variance is decomposed into direct genetic effects, $\sigma_{d}^{2}$, indirect genetic effects of siblings, $\sigma_{i}^{2}$, the covariance between direct and indirect genetic effects, $\sigma_{\mathrm{id}}$, other components making family members similar, $\sigma_{c}^{2}$, and error/unique variance, $\sigma_{\varepsilon }^{2}$.

Indirect genetic effects can be estimated straight from the genotypes of family members of index persons. While genome‐based restricted maximum likelihood (GREML) analysis was originally conceived as a method for estimating single‐nucleotide polymorphism (SNP) heritability (Yang, Lee, Goddard, & Visscher, 2011), that is, the additive effect of an individual's measured or linked to measured common variants, the model was soon expanded by including family members' genotypes to partition this effect into direct genetic effects of the offspring's genotype, and indirect genetic effects of one (Eaves et al., 2014) or both (Eilertsen et al., 2021) parents. The method exploits genetic variance in the population, summarized in genetic relatedness matrices (GRMs) within and between children, mothers, and fathers to describe the distribution of direct and indirect genetic effects across individuals. Here, we adapt GREML to examine indirect genetic effects of siblings by including all phenotyped and genotyped offspring from each family in the analysis (Figure 1). The method is a form of variance component model and provides mechanism‐independent estimates of sibling interactions.

Indirect genetic effects can also be estimated from PGIs, typically for the trait of interest. A class of methods for estimating ‘genetic nurture’, a form of indirect genetic effect, of parents on their children (Kong et al., 2018), has gained widespread application and development (Balbona et al., 2021; Balbona, Kim, & Keller, 2022). The idea is to separate parental alleles transmitted to the offspring from those not transmitted, and then to regress the offspring trait on transmitted and nontransmitted PGIs. The indirect genetic effect is then estimated as a function of the regression coefficient for the nontransmitted PGI, while the direct genetic effect is estimated with the subtraction of the nontransmitted coefficient from the transmitted coefficient. Note that the core of this design is mathematically equivalent to a model where offspring traits are regressed on the parental PGIs and their own PGI (Okbay et al., 2022). This model has previously been generalized to incorporate indirect genetic effects of siblings (Demange et al., 2022; Young et al., 2022).

**Figure 2:**
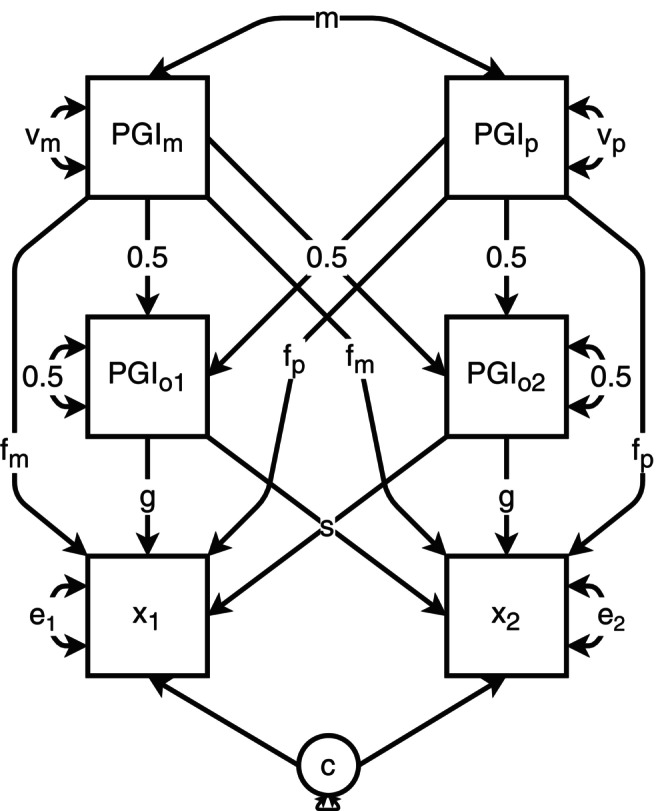
Path diagram of the structural equation model with polygenic indices (SEM‐PGIs), using the conventions of the reticular action model (McArdle, 1980), where two‐headed arrows to endogenous variables denote residuals. Ten parameters are estimated from six observed variables. *g*: the direct genetic effect of an individual's PGI on their own phenotype; *s*: the indirect genetic effects of one sibling's PGI on the other's phenotype; *f*_m/p_: the indirect genetic effect of the mother/father on the offspring's phenotype; the variance of *c*: additional components contributing to phenotypic similarity between siblings; *e*_1/2_: residual variance unique to each sibling; *v*_m/p_: variance of the maternal/paternal genotype; *m*: covariance between maternal and paternal genotypes. The observed variables are PGI_m/p/o1/o2_: polygenic indices of mothers/fathers/sibling 1 and 2, and *x*_1_/*x*_2_: phenotypes of sibling 1 and sibling 2.

Here, we implement this framework using a SEM with full quartet (parents and sibling pair) PGIs as well as phenotypes from the two offspring (SEM‐PGIs; Figure 2). By regressing out the genetic variance shared with parents from offspring genotypes, the path marked *s* (from sibling genotype to index child phenotype) provides an estimate of the indirect genetic effect of one sibling, acting through the traits associated with the PGI, on the phenotype of the other sibling. This method therefore is a form of trait‐based model for estimating indirect genetic effects, similar to models used for examining ‘genetic nurture’ (Balbona et al., 2021, 2022; Kong et al., 2018) that provides estimates of mechanism‐dependent sibling indirect genetic effects.

The absence of sibling interactions is an implicit assumption in genetically informed models of educational performance and mental health (Eilertsen et al., 2021; Howe et al., 2022; Young et al., 2019). We test this assumption empirically using both a variance component and a trait‐based approach. First, we estimate the mechanism‐independent indirect genetic effects of siblings on educational performance and ADHD symptoms using an expansion of the GREML framework. Second, we estimate the trait‐based indirect genetic effects of siblings on the same phenotypes using a sibling SEM‐PGI.

## Methods

### Study sample

All analyses were restricted to participants in the Norwegian Mother, Father, and Child Cohort Study (MoBa; Magnus et al., 2006, Magnus et al., 2016), a population‐based pregnancy cohort study conducted by the Norwegian Institute of Public Health. Pregnant women were recruited from all over Norway in the years 1999–2008. The women consented to participation in 41% of the pregnancies. The cohort includes approximately 114,500 children, 95,200 mothers, and 75,200 fathers, among which 76,577 children, 77,634 mothers, and 53,358 fathers have quality‐controlled genotype data (Corfield et al., 2022). Further, within the genotyped MoBa subsample, there are 41,553 individuals, including 18,152 children, who have a genotyped sibling. All GREML analyses are confined to this subsample of genotyped children with genotyped siblings. Further, MoBa contains 7,132 quartets of genotyped mothers, fathers, and full sibling offspring. All SEM‐PGIs are confined to this genotyped quartets subsample. Finally, while almost all MoBa children have completed compulsory national standardized testing, ADHD symptomatology is available from MoBa questionnaires with less than perfect coverage. Therefore, across methods, analyses of ADHD symptoms are confined to a more restricted sample than the corresponding analysis of educational performance (resulting sample sizes in Tables 1 and 2).

**Table 1:** Results of the full sibling genome‐based restricted maximum likelihood model

	EP 10 years	EP 13 years	EP 14 years	ADHD 3 years	ADHD 8 years
*n*	15,971	15,728	15,426	11,036	7,943
$\sigma_{d}^{2}$	0.266 (0.022)	0.299 (0.022)	0.294 (0.022)	0.086 (0.028)	0.151 (0.043)
$\sigma_{i}^{2}$	0.002 (0.002)	0.007 (0.003)	0.003 (0.002)	0.040 (0.022)	0.001 (0.003)
$\sigma_{\mathrm{id}}$	0.023 (0.011)	0.046 (0.011)	0.029 (0.012)	0.000 (0.021)	−0.011 (0.023)
$\sigma_{c}^{2}$	0.216 (0.025)	0.178 (0.024)	0.193 (0.025)	0.262 (0.037)	0.210 (0.044)
$\sigma_{\varepsilon }^{2}$	0.498 (0.018)	0.471 (0.016)	0.483 (0.017)	0.623 (0.037)	0.650 (0.043)

EP, educational performance. In Norway, this is measured at ages 10, 13, and 14 with compulsory standardized testing. ADHD, attention‐deficit hyperactivity disorder symptoms. Parents in the MoBa cohort are asked to rate their children's behavior with the Child Behavior Checklist at age 3 and the Parent/Teacher Rating Scale for Disruptive Behavior Disorders at age 8. *n*, sample size. $\sigma_{d}^{2}$, variance attributable to direct genetic effects. $\sigma_{i}^{2}$, variance attributable to indirect genetic effects of siblings. $\sigma_{\mathrm{id}}$, covariance between the direct and indirect genetic effects. $\sigma_{c}^{2}$, other sources contributing to sibling similarity, like untagged genetic variance or parental indirect genetic effects. $\sigma_{\varepsilon }^{2}$, variance unique to each individual, often due to random experiences and/or measurement error. Parameters are standardized since the phenotypes are scaled before restricted maximum likelihood estimation. Standard errors are given in parentheses.

**Table 2:** Results of the full structural equation model with polygenic indices

	EP 10 years	EP 13 years	EP 14 years	ADHD 3 years	ADHD 8 years
*n* quartets	7,034	7,001	6,950	5,532	3,990
*g*	0.229 (0.012)	0.254 (0.012)	0.250 (0.013)	0.014 (0.015)	0.090 (0.018)
*s*	0.005 (0.012)	0.010 (0.012)	0.016 (0.013)	−0.029 (0.015)	−0.015 (0.018)
*f*_m_	0.040 (0.014)	0.047 (0.014)	0.050 (0.014)	0.023 (0.017)	−0.001 (0.020)
*f*_p_	0.055 (0.014)	0.072 (0.014)	0.075 (0.014)	0.041 (0.017)	0.002 (0.019)
Var(*c*)	0.360 (0.013)	0.362 (0.012)	0.336 (0.013)	0.346 (0.017)	0.265 (0.021)
*e*_1_	0.555 (0.015)	0.527 (0.014)	0.555 (0.015)	0.652 (0.021)	0.659 (0.026)
*e*_2_	0.553 (0.015)	0.530 (0.014)	0.560 (0.015)	0.649 (0.022)	0.753 (0.028)
*v*_m_	1.003 (0.017)	1.003 (0.017)	0.994 (0.017)	0.988 (0.019)	0.986 (0.022)
*v*_p_	1.000 (0.017)	1.004 (0.017)	1.001 (0.017)	0.997 (0.019)	1.008 (0.023)
*m*	0.123 (0.012)	0.122 (0.012)	0.122 (0.012)	0.035 (0.013)	0.041 (0.016)

EP, educational performance. These models use the educational attainment polygenic index. ADHD, attention‐deficit hyperactivity disorder symptoms. These models use the ADHD polygenic index. *n* quartets, number of mother, father, and two offspring quartets. The structural equation model decomposes the 6 × 6 covariance matrix of the polygenic indices (PGIs) and the offspring phenotypes into parameters *g*, direct genetic effect of an individuals PGI on their own phenotype; *s*, indirect genetic effects of one sibling's PGI on the other's phenotype; *f*_m_, indirect genetic effect of the mother on the offspring's phenotype; *f*_p_, indirect genetic effect of the father on the offspring's phenotype; *c*, additional components contributing to phenotypic similarity between siblings; *e*_1/2_, residual variance unique to each sibling; *v*_m_, variance of the maternal genotype; *v*_p_, variance of the paternal genotype; *m*, covariance between maternal and paternal genotypes. Parameters are standardized since phenotypes are scaled before model fitting. Standard errors are given in parentheses.

### Phenotype data

Since 2007, national standardized testing has been compulsory in Grades 5, 8, and 9 (corresponding to ages ~10, ~13, and ~14) of the Norwegian school system. We access national standardized test results through linkage to Norway's national education database and perform analyses on all measurement points. In Grades 5 and 8, students' skills in reading, mathematics, and English are tested. In Grade 9, skills in reading and mathematics, but not English, are tested. To obtain educational performance scores, we standardize results within subject and year of testing in the full population, then sum across subjects and scale the sum to obtain a zero‐centered distribution with unity variance.

At child ages 1.5, 3, 5, 8, and 14 years, questionnaires containing items relating to ADHD symptoms, among many others, were sent to MoBa participants. Since questionnaire response rates decline with child age, while reliability of neurodevelopmental scales increases, we focus analyses on age 3 and 8. At age 3, ADHD symptoms are parent‐rated using items from the Child Behavior Checklist (Table S1), while at age 8, they are parent‐rated using items from the Parent/Teacher Rating Scale for Disruptive Behavior Disorders (Table S2). The parent responding to the age 3 and age 8 MoBa questionnaires is, with few exceptions, the mother. The six items measured at age 3 and the 18 items measured at age 8 have an internal consistency, indexed with Cronbach's α, of .70 and .91, respectively. Within each age group, items are averaged to provide ADHD symptom scores, which are then scaled in the analytic samples to a zero‐centered distribution with unit variance.

### Genotype data and processing

Blood samples were obtained from both parents during pregnancy and from mothers and children (umbilical cord) at birth (Paltiel et al., 2014). Description of the genotyping, quality control, imputation, and post‐imputation quality control is provided elsewhere (Corfield et al., 2022). Autosomal genotype data passing the MoBaPsychGen pipeline (Corfield et al., 2022) are available from 207,569 individuals of European ancestry and ~7m SNPs.

We calculate a GRM to carry out GREML analyses. First, we select only SNPs with an INFO score above 0.975, meaning we can be highly certain that the imputation is correct, and with a minor allele frequency above 0.01. Then, on all MoBa genotypes, we use the ‐‐make‐grm call in the GCTA software, resulting in a GRM approximating the product of the standardized genotype matrix with its transpose. We subset the resulting GRM by two criteria: (a) having a MoBa genotyped sibling and (b) being registered with the relevant phenotype (i.e. educational performance in Grades 5, 8, or 9, and ADHD symptoms at ages 3 or 8). Finally, to reduce potential conflation of environmental and genetic effects due to cryptic relatedness (relatives outside of the nuclear family can also share aspects of the environment), we filter the resulting GRM by randomly selecting only one individual in nonsibling pairs with relatedness higher than 0.05. To illustrate, this means that from a pair of first cousins once removed, who are expected to have a genetic relatedness of 0.0625, one is excluded, while from a pair of second cousins, who are expected to have a genetic relatedness of 0.03125, both are kept.

We calculate PGIs for educational attainment and ADHD to carry out SEM‐PGIs. PGIs are calculated using LDpred v.1 (Vilhjálmsson et al., 2015) and plink2 (Chang et al., 2015), with MoBa‐excluded summary statistics from GWASes of educational attainment (Okbay et al., 2022) and ADHD (Demontis et al., 2023). First, MoBa genotypes are coordinated with the summary statistics SNPs. Then, weights are linkage disequilibrium adjusted using the European subsample of the 1,000 genomes genotypes (The 1000 Genomes Project Consortium, 2015) as reference panel, and with a prior of 0.5 effective SNPs. Then, PGIs are created using the adjusted weights and the ‐‐score call in plink2. Both educational attainment and ADHD PGIs are residualized against genotyping chip (a factor with six levels), genotyping center (a factor with three levels), and the first 20 principal components of genetic ancestry before analyses are conducted. Finally, quartets in which at least one member had participated in the ADHD GWAS through other projects than MoBa were excluded from analyses with ADHD PGIs (*n* = 28 for symptoms at 3 years, *n* = 21 for symptoms at 8 years).

### Sibling GREML

Sibling GREML is a modification of within‐family GREML models such as M‐GCTA (Eaves et al., 2014) and trio‐GCTA (Eilertsen et al., 2021), allowing for the estimation of indirect genetic effects of siblings. The basic approach is to exploit random genetic covariance in unrelated individuals to associate phenotypic similarity between individuals not only with their own genetic similarity (direct genetic effect) but also with the genetic similarity of their siblings (indirect genetic effect). As an example, when the siblings of unrelated children with similar standardized test scores are themselves likely to be relatively genetically similar over and above family means, this is indicative of a sibling indirect genetic effect on educational performance.

For the *n* × *n* GRM, G, prepared as described in the previous section, there is an *n* × *n* matrix $Z_{i}$ indicating with a one or a zero whether a pair of individuals are siblings or not. We then specify the full GREML model as follows:

$$\mathrm{Cov}\left(y\right)=\sigma_{d}^{2}G+\sigma_{i}^{2}Z_{i}GZ_{i}^{\prime }+\sigma_{\mathrm{id}}\left(Z_{i}G+{\mathrm{GZ}}_{i}^{\prime }\right)+\sigma_{c}^{2}\left(Z_{i}+I\right)+\sigma_{\varepsilon }^{2}Ι$$

where the parameter $\sigma_{d}^{2}$ is the variance of the direct genetic effect on the trait y, $\sigma_{i}^{2}$ is the variance of the indirect genetic effect of siblings, $\sigma_{\mathrm{id}}$ is the covariance between the direct and the indirect genetic effects, and $\mathrm{Cov}\left(y\right)$ is the *n* × *n* covariance matrix for the distribution of y.

Since $Z_{i}+I$ represents the general characteristic of belonging or not belonging to the same family, the estimation of $\sigma_{c}^{2}$ serves the primary purpose of ensuring that estimates of sibling indirect genetic effects do not reflect indirect genetic effects of parents (or other relatives), which are correlated with offspring genotypes. Further, it serves the secondary purpose of ensuring that estimates of sibling indirect genetic effects are not inflated due to siblings, by virtue of sharing genotypes by descent, exhibiting a drastically higher ratio of shared rare to shared common variants than nonrelatives. Finally, the estimation of $\sigma_{\varepsilon }^{2}$ allows individuals to differ for other reasons, such as unique environmental effects. The specification of the sibling GREML model is considered in greater detail in Appendix S1.

### Structural equation model with polygenic indices

The input to the SEM is the covariance matrix between maternal PGI (${\mathrm{PGI}}_{m}$), paternal PGI (${\mathrm{PGI}}_{p}$), younger sibling PGI (${\mathrm{PGI}}_{o_{1}}$), older sibling PGI (${\mathrm{PGI}}_{o_{2}}$), younger sibling phenotype ($x_{1}$), and older sibling phenotype ($x_{2}$). The variances and covariances are modeled as functions of free parameters *v*_m/p_: the variance of parental genotype; *m*: the covariance between maternal and paternal genotype; *g*: the direct genetic effect of offspring genotype on offspring phenotype; *f*_m/p_: the indirect genetic effect of parental genotypes on offspring phenotypes; *s*: the indirect genetic effect of sibling genotype on the other sibling's phenotype; *c*: the shared residual variance among offspring; and *e*: the nonshared residual variance unique to each sibling. Genotype variance as well as indirect genetic effects of parents on offspring could differ between mothers and fathers, so in the implementations of this model, parameters *v*_m/p_ and *f*_m/p_ are allowed to vary by sex (m: maternal; p: paternal) of the parent (Figure 2). Further, parameters fixed to 0.5 include the residual variance of offspring genotype as well as the direct effect of parental genotype on offspring genotype. Then,

$$\mathrm{Cov}\left({\mathrm{PGI}}_{o_{1}},x_{1}\right)=\begin{array}{l} g\frac{2+2m+v_{m}+v_{p}}{4}+s\frac{2m+v_{m}+v_{p}}{4}\\ +\;f_{m}\frac{v_{m}+m}{2}+f_{p}\frac{v_{p}+m}{2}, \end{array}$$

such that the genotype–phenotype covariance in offspring is due to direct genetic effects and indirect genetic effects of both parents and siblings. Thus, the SEM‐PGI approach is a generalization of the fixed effects regression:

$$x_{1j}={g\mathrm{PGI}}_{o_{1}}+s{\mathrm{PGI}}_{o_{2}}+f_{m}{\mathrm{PGI}}_{m}+f_{p}{\mathrm{PGI}}_{p}+c_{j}+e_{1j}$$

$$x_{2j}={g\mathrm{PGI}}_{o_{2}}+s{\mathrm{PGI}}_{o_{1}}+f_{m}{\mathrm{PGI}}_{m}+f_{p}{\mathrm{PGI}}_{p}+c_{j}+e_{2j},$$

where *c* is a random intercept for sibling pair *j*.

### Statistical analyses

We test the significance of parameter estimates by comparing reduced models, where the parameter of interest is fixed to zero, to a fuller model, where this parameter is estimated freely. Under the absence of an effect, the difference in twice the negative log likelihood (Δ‐2LL) between such nested models follows a $\chi^{2}$ distribution with df = Δ*df*, from which *p* values are calculated as the integral from Δ‐2LL and upward. For the SEM‐PGIs, this parameter of interest is the trait‐based sibling indirect genetic effect (s). For the sibling GREML, we initially reduce the model by fixing the direct–indirect covariance parameter ($\sigma_{\mathrm{id}}$) to zero. We then compare this semireduced model to a model where the mechanism‐independent sibling indirect genetic effect ($\sigma_{i}^{2}$) is also fixed to zero (Table S3). Here, the difference between the models is a variance component bounded by zero, and the p value is half that of the ordinary $\chi^{2}$ distribution. Finally, to reduce the potential for false‐negative results on sibling indirect genetic effects, we set α = .05 for each of the 10 models.

## Results

We estimate indirect genetic effects of siblings as well as direct genetic effects on educational performance and ADHD symptoms using two different approaches, one variance component (GREML), accounting for all tagged genetic variance, and one trait‐based (SEM‐PGI), which is limited to previously trait‐associated genetic variance. The variances due to genetic effects within the two approaches are commensurable. In the first, they are obtained directly as estimated parameters, $\sigma_{d}^{2}$ and $\sigma_{i}^{2}$. In the second, they are obtained by multiplying the squared path coefficients, $g^{2}$ and $s^{2}$, with the model‐implied variance of the PGI, $\mathrm{Var}\left({\mathrm{PGI}}_{o}\right)$.

### Sibling indirect genetic effects on educational performance and ADHD symptoms

We did not observe indirect genetic effects of siblings on educational performance at any of the three ages (10, 13, and 14 years) with either the variance component approach ($\sigma_{i}^{2}$ = {0.002, 0.007, 0.003}; Table 1) or the trait‐based approach ($s^{2}\times \mathrm{Var}\left({\mathrm{PGI}}_{o}\right)$ = {0.000, 0.000, 0.000}, Table 2). On the other hand, we observed sibling indirect genetic effects on parent‐rated ADHD symptoms at age 3. Here, the variance component estimate ($\sigma_{i}^{2}$ = 0.040) was statistically significant (*p* = .017) and an order of magnitude greater than the trait‐based estimate ($s^{2}\times \mathrm{Var}\left({\mathrm{PGI}}_{o}\right)$ = 0.001), which did not quite reach significance (*p* = .056). Note, however, that the path coefficient for the trait‐specific sibling indirect genetic effect, *s*, was negative, indicating a contrast effect of sibling ADHD on parent‐rated ADHD symptoms. The observed effect did not persist at age 8 either with the variance component ($\sigma_{i}^{2}$ = 0.001) or the trait‐based model ($s^{2}\times \mathrm{Var}\left({\mathrm{PGI}}_{o}\right)$ = 0.000).

### Direct genetic effects on educational performance and ADHD symptoms

We observed clear direct genetic effects on educational performance at all three ages (10, 13, and 14 years) with both the variance component approach ($\sigma_{d}^{2}$ = {0.266, 0.299, 0.294}, Table 1) and the trait‐based approach ($g^{2}\times \mathrm{Var}\left({\mathrm{PGI}}_{o}\right)$ = {0.056, 0.068, 0.066}, Table 2). Direct genetic effects increased slightly with age and estimates were ~4.5 times greater in GREML than in the SEM‐PGIs. Further, we observed statistically significant direct genetic effects on ADHD symptoms at both ages (3 and 8 years) using the variance component approach ($\sigma_{d}^{2}$ = {0.086, 0.151}), and with the trait‐based approach at age 8 ($g^{2}\times \mathrm{Var}\left({\mathrm{PGI}}_{o}\right)$ = 0.008), but not with the trait‐based approach at age 3 ($g^{2}\times \mathrm{Var}\left({\mathrm{PGI}}_{o}\right)$ = 0.000). Again, direct genetic effects increased with age regardless of model. The difference in direct genetic effect estimates between GREML and SEM‐PGIs was even greater for ADHD symptoms than for educational performance.

### Other effects of belonging to the same family on educational performance and ADHD symptoms

Siblings can be similar for other reasons than sharing tagged or trait‐associated genetic variance or due to sibling indirect genetic effects. First, they can share untagged genetic variance or non‐trait‐associated genetic variance. Second, they can share indirect genetic effects of other relatives like parents. Third, they can share other environmental features, such as school quality, which may or may not be associated with their genotypes. The $\sigma_{c}^{2}$ component of the sibling GREML and the c component of the sibling SEM‐PGI differ in the extent to which they reflect these different variance sources. All of these sources are captured by the $\sigma_{c}^{2}$ component, with the crucial exception of tagged but non‐trait‐associated genetic variance, which GREML recognizes as genetic effects. All of these sources are also captured by the c component, except parental trait‐associated indirect genetic effects, which are estimated explicitly in the SEM‐PGI. The $\sigma_{c}^{2}$ component is smaller than the variance of the c component for all analyzed traits, including educational performance at 10 years ($\sigma_{c}^{2}$ = 0.216 vs. $\mathrm{Var}\left(c\right)$ = 0.360), 13 years ($\sigma_{c}^{2}$ = 0.178 vs. $\mathrm{Var}\left(c\right)$ = 0.362), and 14 years ($\sigma_{c}^{2}$ = 0.193 vs. $\mathrm{Var}\left(c\right)$ = 0.336), as well as ADHD symptoms at 3 years ($\sigma_{c}^{2}$ = 0.262 vs. $\mathrm{Var}\left(c\right)$ = 0.346) and 8 years ($\sigma_{c}^{2}$ = 0.210 vs. $\mathrm{Var}\left(c\right)$ = 0.266).

Part of the reason for this is that the variance due to trait‐specific indirect genetic effects of parents, estimated as the product of the squared parental path coefficient ($f_{m}^{2}$ or $f_{p}^{2}$) and the variance of the parental PGI ($v_{m}$ or $v_{p}$), is small across analyzed traits. This includes educational performance at age 10 ($f_{m}^{2}\times v_{m}$ = 0.002, $f_{p}^{2}\times v_{p}$ = 0.003), age 13 ($f_{m}^{2}\times v_{m}$ = 0.002, $f_{p}^{2}\times v_{p}$ = 0.005), and age 14 ($f_{m}^{2}\times v_{m}$ = 0.002, $f_{p}^{2}\times v_{p}$ = 0.006), and especially for ADHD symptoms at age 3 ($f_{m}^{2}\times v_{m}$= 0.001, $f_{p}^{2}\times v_{p}$ = 0.002) and age 8 ($f_{m}^{2}\times v_{m}$ = 0.000, $f_{p}^{2}\times v_{p}$ = 0.000). Notably, indirect genetic effects of fathers are consistently greater than indirect genetic effects of mothers. Further, fixing the sibling indirect effect parameter for ADHD symptoms at age 3 to zero results in reduced estimates for parental indirect genetic effects ($f_{m}^{2}\times v_{m}$ = 0.000, $f_{p}^{2}\times v_{p}$ = 0.001; Table S4).

## Discussion

Over the last decade, molecular genetic research has increasingly made use of within‐family designs to decompose genotype–trait covariances into direct and indirect genetic effects (Cheesman et al., 2023). We show that a well‐known genetically informative model (Eaves et al., 2014; Eilertsen et al., 2021; Yang et al., 2011) can be tweaked to include estimates of sibling indirect genetic effects. The resulting approach, sibling GREML, decomposes offspring phenotypic variance into direct genetic effects of their own genome, indirect genetic effects of their sibling's genome, covariances between the direct and indirect genetic effects, and other sources of sibling similarity. It is a variance component approach that disregards specific mechanisms through which either direct or indirect genetic effects might operate and is therefore mechanism‐independent. Sibling GREML also permits multiple offspring from the same family to serve as both predicting and predicted sibling at once. In addition to increasing power, this produces estimates of sibling indirect genetic effects that are multidirectional rather than constrained to a particular direction like older to younger. The second model, sibling SEM‐PGI, corresponds to previously presented fixed effects models including sibling genotypes (Demange et al., 2022; Young et al., 2022). It extends a statistical framework mathematically equivalent (Okbay et al., 2022) to the nontransmitted alleles design (Kong et al., 2018) by estimating direct genetic effects, sibling indirect genetic effects, parental indirect genetic effects, and other sources of sibling similarity, along with parental genetic variances and covariances. It is a trait‐based approach which provides estimates of genetic effects operating through mechanisms associated with the particular PGIs used and is therefore mechanism‐dependent.

Indirect genetic effects of siblings are only observed for ADHD symptoms at 3 years and not for educational performance at 10, 13, or 14 years, or for ADHD symptoms at 8 years. The variance component estimate of sibling indirect genetic effects on ADHD symptoms at 3 years is an order of magnitude greater than the trait‐based estimate. This is due to an unknown mix of (a) genetic variance contributing to ADHD symptom variance but not captured by the ADHD PGI and (b) the presence of mechanism‐independent sibling indirect genetic effects operating through phenotypic processes not associated with the ADHD PGI. This latter possibility is crucial. Like‐to‐like mechanisms occur when the trait of an index child is influenced by the same trait in a sibling. The possibility suggests that some of the mechanisms through which family members influence each other are not like‐to‐like.

With the development of within‐family models to decompose gene–trait associations into direct genetic effects and parental indirect genetic effects, there has been some concern, among researchers, about the validity of the typical underlying assumption of no sibling indirect genetic effects (Demange et al., 2022; Howe et al., 2022). The absence of both mechanism‐dependent and mechanism‐independent sibling indirect genetic effects on educational performance observed in the present study, although no guarantee for unbiased direct and parental indirect genetic effects estimates in other samples, could still be taken as a reassurance for researchers restricting their within‐family studies to trios. Further, indirect genetic effects of other types of relatives might be more important than siblings in the context of educational performance (Nivard et al., 2024).

The path coefficient for the trait‐based sibling indirect genetic effect on parent‐rated ADHD symptoms at 3 years is negative, meaning that higher genetic liability to ADHD in one sibling decreases parent‐rated ADHD symptoms in the other sibling. Previously, negative covariances between indirect and direct genetic effects on conduct and inattention problems in children have suggested that families might create environments that counteract the genetic propensities of family members (Eilertsen et al., 2022). Further, analogous contrast effects of siblings on parent‐rated ADHD symptoms have been observed previously in the quantitative genetics literature (Boomsma et al., 2005; Rietveld et al., 2003; Rietveld, Hudziak, Bartels, van Beijsterveldt, & Boomsma, 2004; Simonoff et al., 1998). Notably, patterns of low or even negative dizygotic twin correlations in parent‐rated ADHD symptoms, which have been interpreted as possible sibling interaction effects (Boomsma et al., 2005), were not found in different teacher‐rated ADHD symptoms, with the implication that they originate in parental rating bias rather than in effects of one sibling's ADHD symptoms on the other's true ADHD symptom levels (Simonoff et al., 1998).

Although we do not have different teacher‐rated ADHD symptoms to compare, it is plausible that the sibling indirect genetic effects observed in the present study are also at least in part a consequence of parental rating bias. The sibling indirect genetic effects are observed only for the parent‐rated trait, and only at 3 years old, when parents might be more likely to let supportive information like sibling behavior anchor their judgments pending stable or clear behavior patterns in the index child. The low variance component and absent trait‐based direct genetic effects on the same phenotype might support this interpretation. If the sibling genotype influences only the parental rating and not the actual behavior of the index child, it is strictly speaking an indirect genetic effect on the parent. Nevertheless, this effect of one offspring on the parent could still have consequences for the other offspring, for example, if the parent's altered judgment influences their inclination to seek medical attention on behalf of their child. Further, even if the effect were purely due to parental rating bias, it could also still influence other parameters estimated in within‐family models. For example, in the SEM‐PGI of ADHD symptoms at 3 years, we observe that parental indirect genetic effects are suppressed when the sibling indirect genetic effect is not explicitly modeled.

Direct genetic effects are observed for all traits, including educational performance at ages 10, 13, and 14, as well as ADHD symptoms at ages 3 and 8. The trait variance due to direct genetic effects as estimated with the sibling GREML model is always much larger than the trait variance due to direct genetic effects as estimated with the sibling SEM‐PGI, to the point where a clear variance component direct genetic effect on ADHD symptoms at age 3 is not recovered using the trait‐based approach. Here, these differences are completely due to genetic variances contributing to trait variance but not captured by the educational attainment or the ADHD PGIs. Further, both variance component and trait‐based direct genetic effect estimates on both educational performance and ADHD symptoms increase with age, in line with previously observed age‐increasing heritabilities (Haworth et al., 2010).

We have applied two within‐family models, one GREML and one SEM‐PGI, to estimate sibling indirect genetic effects and thereby reduce potential bias from this source, if present, on estimates of direct genetic effects and parental indirect genetic effects. Naturally, extensions to siblings come with their own set of violable assumptions. In the sibling GREML, we include as many siblings as possible from each family in the model, at times amounting to more than two, with the implication that the genotypes of each additional sibling will contribute equally to an index child's phenotypic variance. The additivity of potential sibling indirect genetic effects should be addressed in future research. Genetic effects between relatives are likely to be correlated and are inherently difficult to disentangle; however, the addition of exogenous genetic variation from siblings allows for more accurate parameter estimates.

## Conclusion

We do not observe any impact of sibling genotypes on children's educational performance at ages 10, 13, and 14, or their ADHD symptoms at age 8. Children's ADHD symptoms at age 3, on the other hand, are contrastingly impacted – a weak effect that might reflect parental rating bias. These results suggest that within‐family models of educational performance are unlikely to be drastically biased by an assumption of absent sibling indirect genetic effects. We suggest that analyses of indirect genetic effects benefit from combining trait‐based and variance component approaches.

## Ethical considerations

The establishment of MoBa and initial data collection was based on a license from the Norwegian Data Protection Agency and approval from the Regional Committees for Medical and Health Research Ethics. The MoBa cohort is currently regulated by the Norwegian Health Registry Act. MoBa is based on informed consent from all participants in accordance with research ethical guidelines. The processing of data for MoBa participants and their relatives is based on public interest and scientific research purposes in accordance with the General Data Protection Regulation (GDPR) Section 6e and Section 9j and the Norwegian Personal Data Act Sections 8 and 9. The Norwegian registry and MoBa data used were from the project SUBPU. The Department of Psychology, University of Oslo, is responsible for the data handling of SUBPU; a Data Protection Impact Assessment (DPIA) has been signed by the head of department, and the project manager is E.Y. SUBPU was approved by the Regional Committee for Medical and Health Research Ethics (REK sør‐øst) on May 7, 2018 (Ref. #2017/2205). SUBPU has agreements with MoBa and Statistics Norway for data linkage and usage.

**Key points**

**What's known?**

- Within‐family molecular genetic models of child development are increasingly used, and typically assume that indirect genetic effects are restricted to parents.

**What's new?**

- We expand two molecular genetic designs, genome‐based restricted maximum likelihood and structural equation modeling with polygenic indices, to also model indirect genetic effects of siblings on the development of attention‐deficit hyperactivity disorder (ADHD) symptoms and educational performance in Norwegian children.
- With both approaches and across phenotypes, indirect genetic effects of siblings are negligible.

**What's relevant?**

- Trio and within‐sibship models of educational performance are unlikely to be drastically biased by sibling indirect genetic effects.

## Supporting information


**Table S1.** Child Behavior Checklist. Six items indexing attention problems/hyperactivity. Measured at 3 years.
**Table S2.** Parent/Teacher Rating Scale for Disruptive Behavior Disorders (RS‐DBD). Eighteen items related to attention‐deficit hyperactivity disorder (ADHD). Measured at 8 years.
**Table S3.** Genome‐based restricted maximum likelihood (GREML) results.
**Table S4.** Structural equation model with polygenic indices results.
**Appendix S1.** Sibling genome‐based restricted maximum likelihood, details on the genetic components.

## Data Availability

Data from the Norwegian Mother, Father and Child Cohort Study and the Medical Birth Registry of Norway used in this study are managed by the national health register holders in Norway (Norwegian Institute of Public Health) and can be made available to researchers, provided approval from the Regional Committees for Medical and Health Research Ethics (REC), compliance with the EU General Data Protection Regulation (GDPR), and approval from the data owners. The consent given by the participants does not open for storage of data on an individual level in repositories or journals. SEM‐PGI covariance matrices are available at https://github.com/mathiava/sibIGE. Researchers who want access to data sets for replication should apply through helsedata.no. Access to data sets requires approval from the Regional Committee for Medical and Health Research Ethics in Norway and an agreement with MoBa. The code used for sibling GREML and sibling SEM‐PGI is available at https://github.com/mathiava/sibIGE.
