# Supplementary material for: Indirect genetic effects of siblings
Source: J Child Psychol Psychiatry. 2026 Feb 12;67(8):1269–79. doi: 10.1111/jcpp.70130 (PMC13341396; doi:10.1111/jcpp.70130)
Supplement: Supplementary file 1 — Table S1. Child Behavior Checklist. Six items indexing attention problems/hyperactivity. Measured at 3 years. Table S2. Parent/Teacher Rating Scale for Disruptive Behavior Disorders (RS‐DBD). Eighteen items related to attention‐deficit hyperactivity disorder (ADHD). Measured at 8 years. Table S3. Genome‐based restricted maximum likelihood (GREML) results. Table S4. Structural equation model with polygenic indices results. Appendix S1. Sibling genome‐based restricted maximum likelihood, details on the genetic components. [file JCPP-67-1269-s001.docx]

**Indirect genetic effects of siblings**

**Supporting Information**

**Table S1.** Child Behaviour Checklist. 6 items indexing attention problems/hyperactivity. Measured at 3 years.

| **Item** | **Value** | **Distribution** |
| --- | --- | --- |
| 2. Can’t concentrate, can’t pay attention for long | 1 – Not true  2 – Somewhat or sometimes true  3 – Very true or often true |  |
| 3. Can’t sit still, restless or overactive |  |  |
| 3. Can’t stand waiting, wants everything now |  |  |
| 8. Demands must be met immediately |  |  |
| 15. Gets into everything |  |  |
| 20. Quickly shifts from one activity to another |  |  |
| CBCL attention problems/hyperactivity sumscore |  |  |

**Table S2.** Parent/Teacher Rating Scale for Disruptive Behaviour Disorders (RS-DBD). 18 items related to ADHD. Measured at 8 years.

| **Item** | **Value** | **Distribution** |
| --- | --- | --- |
| 1. Fails to give close attention to details or makes careless mistakes in schoolwork | 1 – Never/rarely  2 – Sometimes  3 – Often  4 – Very often |  |
| 2. Has difficulty sustaining attention in tasks or play activities |  |  |
| 3. Does not seem to listen when spoken to directly |  |  |
| 4. Does not follow through on instructions and fails to finish school work, chores or duties (not due to oppositional behaviour or failure to understand instructions) |  |  |
| 5. Has difficulty organizing tasks and activities |  |  |
| 6. Avoids, dislikes or is reluctant to engage in tasks that require sustained mental effort (such as schoolwork or homework) |  |  |
| 7. Loses things necessary for tasks or activities (pencils, books, toys) |  |  |
| 8. Is easily distracted |  |  |
| 9. Is forgetful in faily activities |  |  |
| 10. Fidgets with hands or feet or squirms in seat (sits uneasily) |  |  |
| 11. Leaves seat in classroom or in other situations in which remaining seated is expected (e.g. at the table or in group gathering) |  |  |
| 12. Runs about or climbs excessively in situations in which it is inappropriate |  |  |
| 13. Has difficulty playing or engaging in leisure activities quietly |  |  |
| 14. Is “on the go” or acts as if “driven by a motor” |  |  |
| 15. Talks excessively |  |  |
| 16. Blurts out answers before questions have been completed |  |  |
| 17. Has difficulty awaiting turn |  |  |
| 18. Interrupts or intrudes on others, such as in conversation or play |  |  |
| RS-DBD ADHD symptoms sumscore |  |  |

**Table S3.** GREML results

|  | *EP 10 yrs* | *EP 13 yrs* | *EP 14 yrs* | *ADHD 3yrs* | *ADHD 8yrs* |
| --- | --- | --- | --- | --- | --- |
| n | 15,971 | 15,728 | 15,426 | 11,036 | 7,943 |
|  | ***Full model*** | | | | |
| $\boldsymbol{\sigma}_{\boldsymbol{d}}^{\boldsymbol{2}}$ | 0.266 (0.022) | 0.299 (0.022) | 0.294 (0.022) | 0.086 (0.028) | 0.151 (0.043) |
| $\boldsymbol{\sigma}_{\boldsymbol{i}}^{\boldsymbol{2}}$ | 0.002 (0.002) | 0.007 (0.003) | 0.003 (0.002) | 0.040 (0.022) | 0.001 (0.003) |
| $\boldsymbol{\sigma}_{\boldsymbol{id}}$ | 0.023 (0.011) | 0.046 (0.011) | 0.029 (0.012) | 0.000 (0.021) | -0.011 (0.023) |
| $\boldsymbol{\sigma}_{\boldsymbol{c}}^{\boldsymbol{2}}$ | 0.216 (0.025) | 0.178 (0.024) | 0.193 (0.025) | 0.262 (0.037) | 0.210 (0.044) |
| $\boldsymbol{\sigma}_{\boldsymbol{\varepsilon}}^{\boldsymbol{2}}$ | 0.498 (0.018) | 0.471 (0.016) | 0.483 (0.017) | 0.623 (0.037) | 0.650 (0.043) |
|  | ***Comparison between full and semi-reduced model*** | | | | |
| Δ-2LL | 3.919 | 15.965 | 6.024 | 0.000 | 0.251 |
| p | 0.047 | 6.45×10^-5^ | 0.014 | 0.983 | 0.616 |
|  | ***Semi-reduced model:*** $\boldsymbol{\sigma}_{\boldsymbol{id}}\boldsymbol{=0}$ | | | | |
| $\boldsymbol{\sigma}_{\boldsymbol{d}}^{\boldsymbol{2}}$ | 0.275 (0.022) | 0.310 (0.022) | 0.303 (0.022) | 0.086 (0.028) | 0.145 (0.041) |
| $\boldsymbol{\sigma}_{\boldsymbol{i}}^{\boldsymbol{2}}$ | 0.000 (1.28×10^-9^) | 0.006 (0.013) | 0.000 (3.46×10^-5^) | 0.040 (0.018) | 0.000 (1.28×10^-5^) |
| $\boldsymbol{\sigma}_{\boldsymbol{c}}^{\boldsymbol{2}}$ | 0.255 (0.016) | 0.253 (0.017) | 0.242 (0.016) | 0.261 (0.022) | 0.193 (0.027) |
| $\boldsymbol{\sigma}_{\boldsymbol{\varepsilon}}^{\boldsymbol{2}}$ | 0.473 (0.013) | 0.424 (0.013) | 0.452 (0.013) | 0.623 (0.020) | 0.666 (0.027) |
|  | ***Comparison between semi-reduced and reduced model*** | | | | |
| Δ-2LL | 0.000 | 0.252 | 0.000 | 5.745 | 0.000 |
| p | 0.500 | 0.307 | 0.500 | 0.009 | 0.500 |
|  | ***Reduced model:*** $\boldsymbol{\sigma}_{\boldsymbol{i}}\boldsymbol{=}\boldsymbol{\sigma}_{\boldsymbol{id}}\boldsymbol{=0}$ | | | | |
| $\boldsymbol{\sigma}_{\boldsymbol{d}}^{\boldsymbol{2}}$ | 0.275 (0.022) | 0.310 (0.022) | 0.303 (0.022) | 0.090 (0.028) | 0.145 (0.041) |
| $\boldsymbol{\sigma}_{\boldsymbol{c}}^{\boldsymbol{2}}$ | 0.255 (0.016) | 0.257 (0.016) | 0.242 (0.016) | 0.278 (0.021) | 0.193 (0.027) |
| $\boldsymbol{\sigma}_{\boldsymbol{\varepsilon}}^{\boldsymbol{2}}$ | 0.473 (0.013) | 0.426 (0.013) | 0.452 (0.013) | 0.635 (0.019) | 0.666 (0.027) |

Extension of Table 1, with parameter estimates from semi-reduced models, where $\sigma_{id}$ is fixed to zero, and reduced models, where both $\sigma_{id}$and $\boldsymbol{\sigma}_{\boldsymbol{i}}^{\boldsymbol{2}}$ are fixed to zero. **Δ-2LL**: The difference in -2loglikelihood between nested models. **p**: p-value of the -2loglikelihood difference. For the evaluation of sibling indirect genetic effects, the semi-reduced model is compared to the reduced model.

**Table S4.** Structural equation model with polygenic indices results.

|  | *EP 10 yrs* | *EP 13 yrs* | *EP 14 yrs* | *ADHD 3yrs* | *ADHD 8yrs* |
| --- | --- | --- | --- | --- | --- |
| n quartets | 7034 | 7001 | 6950 | 5532 | 3990 |
|  | ***Full model*** | | | | |
| *g* | 0.229 (0.012) | 0.254 (0.012) | 0.250 (0.013) | 0.014 (0.015) | 0.090 (0.018) |
| *s* | 0.005 (0.012) | 0.010 (0.012) | 0.016 (0.013) | -0.029 (0.015) | -0.015 (0.018) |
| *f_m_* | 0.040 (0.014) | 0.047 (0.014) | 0.050 (0.014) | 0.023 (0.017) | -0.001 (0.020) |
| *f_p_* | 0.055 (0.014) | 0.072 (0.014) | 0.075 (0.014) | 0.041 (0.017) | 0.002 (0.019) |
| *Var(c)* | 0.360 (0.013) | 0.362 (0.012) | 0.336 (0.013) | 0.346 (0.017) | 0.265 (0.021) |
| *e_1_* | 0.555 (0.015) | 0.527 (0.014) | 0.555 (0.015) | 0.652 (0.021) | 0.659 (0.026) |
| *e_2_* | 0.553 (0.015) | 0.530 (0.014) | 0.560 (0.015) | 0.649 (0.022) | 0.753 (0.028) |
| *v_m_* | 1.003 (0.017) | 1.003 (0.017) | 0.994 (0.017) | 0.988 (0.019) | 0.986 (0.022) |
| *v_p_* | 1.000 (0.017) | 1.004 (0.017) | 1.001 (0.017) | 0.997 (0.019) | 1.008 (0.023) |
| *m* | 0.123 (0.012) | 0.122 (0.012) | 0.122 (0.012) | 0.035 (0.013) | 0.041 (0.016) |
|  | ***Comparison between full and reduced model*** | | | | |
| Δ-2LL | 0.158 | 0.702 | 1.630 | 3.592 | 0.667 |
| p | 0.691 | 0.402 | 0.202 | 0.058 | 0.414 |
|  | ***Reduced model:*** $\boldsymbol{s=0}$ | | | | |
| *g* | 0.227 (0.012) | 0.250 (0.012) | 0.245 (0.012) | 0.021 (0.015) | 0.092 (0.018) |
| *f_m_* | 0.043 (0.011) | 0.054 (0.011) | 0.061 (0.012) | 0.005 (0.014) | -0.010 (0.016) |
| *f_p_* | 0.058 (0.011) | 0.079 (0.011) | 0.086 (0.012) | 0.023 (0.014) | -0.006 (0.016) |
| *Var(c)* | 0.360 (0.013) | 0.362 (0.012) | 0.336 (0.013) | 0.346 (0.017) | 0.265 (0.021) |
| *e_1_* | 0.555 (0.015) | 0.527 (0.014) | 0.555 (0.015) | 0.653 (0.021) | 0.660 (0.026) |
| *e_2_* | 0.553 (0.015) | 0.530 (0.014) | 0.560 (0.015) | 0.649 (0.022) | 0.753 (0.028) |
| *v_m_* | 1.003 (0.017) | 1.003 (0.017) | 0.994 (0.017) | 0.988 (0.019) | 0.986 (0.022) |
| *v_p_* | 1.000 (0.017) | 1.004 (0.017) | 1.001 (0.017) | 0.997 (0.019) | 1.008 (0.023) |
| *m* | 0.123 (0.012) | 0.122 (0.012) | 0.122 (0.012) | 0.035 (0.013) | 0.041 (0.016) |

Extension of Table 2, with parameter estimates from the reduced model, where *s* is fixed to zero. **Δ-2LL**: The difference in -2loglikelihood between nested models. **p**: p-value of the -2loglikelihood difference. For the evaluation of sibling indirect genetic effects, the full model is compared to the reduced model.

**Appendix S1.** Sibling genome-based restricted maximum likelihood, details on the genetic components.

As an illustration, with two sibling pairs (1, 3) and (2, 4), a model for direct genetic effects, *d*, and sibling indirect genetic effects, *i*, on a trait *y*, is:

$$\left[ \begin{matrix} y_{1} \\ y_{2} \\ y_{3} \\ y_{4} \end{matrix} \right]= \left[ \begin{matrix} 1 & 0 & 0 & 0 \\ 0 & 1 & 0 & 0 \\ 0 & 0 & 1 & 0 \\ 0 & 0 & 0 & 1 \end{matrix} \right]\left[ \begin{matrix} d_{1} \\ d_{2} \\ d_{3} \\ d_{4} \end{matrix} \right]+\left[ \begin{matrix} 0 & 0 & 1 & 0 \\ 0 & 0 & 0 & 1 \\ 1 & 0 & 0 & 0 \\ 0 & 1 & 0 & 0 \end{matrix} \right]\left[ \begin{matrix} i_{1} \\ i_{2} \\ i_{3} \\ i_{4} \end{matrix} \right]= \left[ \begin{matrix} d_{1} \\ d_{2} \\ d_{3} \\ d_{4} \end{matrix} \right]+\left[ \begin{matrix} i_{3} \\ i_{4} \\ i_{1} \\ i_{2} \end{matrix} \right]$$

In general,

$$\boldsymbol{y=}\left[ \begin{matrix} \boldsymbol{Z}_{d} & \boldsymbol{Z}_{i} \end{matrix} \right]\left[ \begin{matrix} \boldsymbol{d} \\ \boldsymbol{i} \end{matrix} \right]$$

where $\boldsymbol{Z}_{d}$ is the identity matrix and $\boldsymbol{Z}_{i}$ is the matrix indicating with a one or a zero whether a pair of individuals are siblings or not. With $\boldsymbol{G}$ being the GRM, the covariance matrix between direct and indirect genetic effects can be described as

$Cov\left[ \begin{matrix} \boldsymbol{d} \\ \boldsymbol{i} \end{matrix} \right]\boldsymbol{=}\left[ \begin{matrix} \sigma_{d}^{2}\boldsymbol{G} & \sigma_{id}\boldsymbol{G} \\ \sigma_{id}\boldsymbol{G} & \sigma_{i}^{2}\boldsymbol{G} \end{matrix} \right]$,

such that the genetic component of the marginal distrubution of the trait is

$Cov\boldsymbol{(y)=}\left[ \begin{matrix} \boldsymbol{Z}_{d} & \boldsymbol{Z}_{i} \end{matrix} \right]\left[ \begin{matrix} \sigma_{d}^{\boldsymbol{2}}\boldsymbol{G} & \sigma_{id}\boldsymbol{G} \\ \sigma_{id}\boldsymbol{G} & \sigma_{i}^{2}\boldsymbol{G} \end{matrix} \right]\left[ \begin{matrix} \boldsymbol{Z}_{d}^{\boldsymbol{'}} \\ \boldsymbol{Z}_{i}^{\boldsymbol{'}} \end{matrix} \right]$,

i.e.

$Cov\left( \boldsymbol{y} \right)\boldsymbol{=}\sigma_{d}^{2}\boldsymbol{G}{\boldsymbol{+}\sigma_{i}^{2}\boldsymbol{Z}}_{\boldsymbol{i}}\boldsymbol{G}\boldsymbol{Z}_{\boldsymbol{i}}^{\boldsymbol{'}}\boldsymbol{+}\sigma_{id}\left( \boldsymbol{Z}_{\boldsymbol{i}}\boldsymbol{G+}\boldsymbol{GZ}_{\boldsymbol{i}}^{\boldsymbol{'}} \right)$.

To flesh out the matrices involved in this expression, consider again our two sibling pairs (1, 3) and (2, 4), for which

$\boldsymbol{G}=\left[ \begin{matrix} a_{11} & a_{12} & a_{13} & a_{14} \\ a_{21} & a_{22} & a_{23} & a_{24} \\ a_{31} & a_{32} & a_{33} & a_{34} \\ a_{41} & a_{42} & a_{43} & a_{44} \end{matrix} \right]$

and, as we know,

$\boldsymbol{Z}_{\boldsymbol{i}}\boldsymbol{=}\left[ \begin{matrix} 0 & 0 & 1 & 0 \\ 0 & 0 & 0 & 1 \\ 1 & 0 & 0 & 0 \\ 0 & 1 & 0 & 0 \end{matrix} \right]$.

Then,

$\boldsymbol{Z}_{\boldsymbol{i}}\boldsymbol{G}\boldsymbol{Z}_{\boldsymbol{i}}^{\boldsymbol{'}}\boldsymbol{=}\left[ \begin{matrix} a_{33} & a_{34} & a_{31} & a_{32} \\ a_{43} & a_{44} & a_{41} & a_{42} \\ a_{13} & a_{14} & a_{11} & a_{12} \\ a_{23} & a_{24} & a_{21} & a_{22} \end{matrix} \right]$,

such that, in the model specified above, the phenotypic similarity of two individuals is expressed in part by the genotypic similarity of their siblings. This is the sibling indirect genetic effect. Further,

$\boldsymbol{Z}_{\boldsymbol{i}}\boldsymbol{G+}\boldsymbol{GZ}_{\boldsymbol{i}}^{\boldsymbol{'}}\boldsymbol{=}\left[ \begin{matrix} a_{13}+ a_{31} & a_{14}+ a_{32} & a_{11}+ a_{33} & a_{12}+ a_{34} \\ a_{23}+ a_{41} & a_{24}+ a_{42} & a_{21}+ a_{43} & a_{22}+ a_{44} \\ a_{33}+ a_{11} & a_{34}+ a_{12} & a_{31}+ a_{13} & a_{32}+ a_{14} \\ a_{43}+ a_{21} & a_{44}+ a_{22} & a_{41}+ a_{23} & a_{42}+ a_{24} \end{matrix} \right]$,

such that, in the specified model, the phenotypic similarity of two individuals i,j is also expressed in part by the genotypic similarity between i and the sibling of j plus the genotypic similarity between j and the sibling of i. This is the covariance between direct and indirect genetic effects.
